# The Role of microRNA in Gastric Malignancy

**DOI:** 10.3390/ijms14059487

**Published:** 2013-04-29

**Authors:** Toshihiro Nishizawa, Hidekazu Suzuki

**Affiliations:** 1 Division of Gastroenterology and Hepatology, Department of Internal Medicine, Keio University School of Medicine, 35 Shinanomachi, Shinjuku-ku, Tokyo 160-8582, Japan; E-Mail: nisizawa@kf7.so-net.ne.jp; 2 Division of Gastroenterology, National Hospital Organization Tokyo Medical Center, 2-5-1 Higashigaoka, Meguro-ku, Tokyo 152-8902, Japan

**Keywords:** microRNA, *Helicobacter pylori*, gastric cancer

## Abstract

*Helicobacter pylori* (*H. pylori*) infection is the main cause of gastritis, gastro-duodenal ulcer, and gastric cancer. MicroRNAs (miRNAs) are small noncoding RNAs that function as endogenous silencers of numerous target genes. Many miRNA genes are expressed in a tissue-specific manner and play important roles in cell proliferation, apoptosis, and differentiation. Recent discoveries have shed new light on the involvement of miRNAs in gastric malignancy. However, at the same time, several miRNAs have been associated with opposing events, leading to reduced inflammation, inhibition of malignancy, and increased apoptosis of transformed cells. The regulation of miRNA expression could be a novel strategy in the chemoprevention of human gastric malignancy. In this article, the biological importance of miRNAs in gastric malignancy is summarized.

## 1. Introduction

*Helicobacter pylori* (*H. pylori*) infection is one of the most prevalent infectious diseases worldwide, and is estimated to affect 40%–50% of the world population [1]. *H. pylori* has been identified as a group 1 carcinogen by the World Health Organization and is associated with the development of gastric cancer [2]. *H. pylori* eradication has been shown to have a prophylactic effect on gastric cancer [3,4].

MicroRNAs (miRNAs) are noncoding RNAs comprising 18–24 nucleotides that can post-transcriptionally downregulate various target genes [5]. It is estimated that the human genome encodes more than one thousand miRNAs, targeting 30%–60% of all protein-coding genes [6]. They are expressed in a tissue-specific manner, and play important roles in cell proliferation, apoptosis, and differentiation [5,7]. Moreover, recent studies have shown a connection between aberrant expression of miRNAs and the development of cancer. In this article, the biological importance of miRNAs in gastric malignancy is summarized.

## 2. *Helicobacter pylori* and miRNA

CagA of *H. pylori* is a bacterium-derived oncogenic protein closely associated with the development of gastric cancers [8]. After injection into host cells using a type IV secretion system, CagA is phosphorylated at tyrosine residues by the c-Src and Lyn kinases. Phosphorylated CagA then activates the Src homology-2 domain-containing phosphatase 2 (SHP2), which activates the Erk1/2 pathway. CagA translocated into CD44v9-positive gastric cancer stem-like cells is thought to escape from reactive oxygen species-dependent autophagy, resulting in gastric carcinogenesis [9].

miRNA changes in response to *H. pylori* infection are summarized in Table 1. *miR-584* and *miR-1290* expression are upregulated in CagA-transformed cells. The *miR-584* and *miR-1290* target is Foxa1, and knockdown of Foxa1 promotes the epithelial-mesenchymal transition (EMT). Overexpression of *miR-584* and *miR-1290* induces intestinal metaplasia of gastric epithelial cells. These results indicate that *miR-584* and *miR-1290* interfere with cell differentiation and lead to remodeling of the gastric mucosal tissues [10].

CagA enhances *c-myc* and DNA methyltransferase 3B, and attenuates *miR-6a* and *miR-101* expression, which results in the attenuation of *let-7* expression by histone and DNA methylation. CagA induces aberrant epigenetic silencing of *let-7* expression, leading to Ras pathway activation. Thus, the miRNA pathway is a new pathogenic mechanism for CagA.

Shiotani *et al.* [11] reported that the expression of oncogenic miRNAs (*miR-17/92* and the *miR-106b-93-25* cluster, *miR-21, miR-194*, and *miR-196*) is significantly higher in the intestinal metaplasia than in the non-intestinal metaplasia. *H. pylori* eradication improves miRNA deregulation, but not in the intestinal metaplasia. Long-term colonization of *H. pylori* might induce an epigenetic modification of gastric mucosal genes, including the promoter of tumor suppressor miRNAs, but this is not completely reversible by bacterial eradication alone. Epigenetic therapy in severe atrophic or metaplastic gastric mucosa after *H. pylori* eradication might be a possible option for gastric cancer prevention.

## 3. Cell Cycle Progression and miRNA

**Figure 1:**
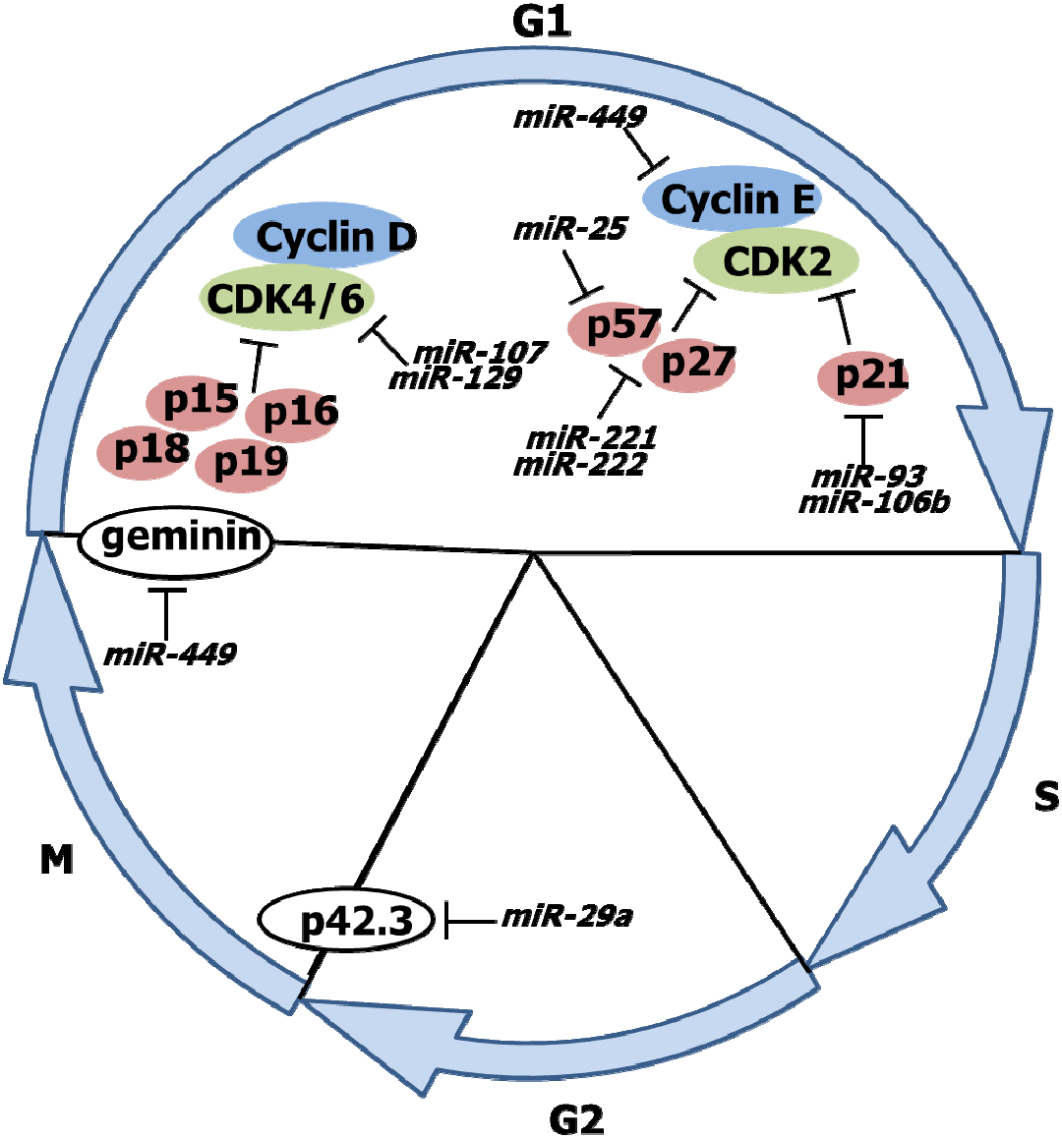
Regulation of cell cycle progression. Cyclins and cyclin-dependent kinases (CDKs) determine a cell’s progress through the cell cycle.

The dysregulation of cell cycle progression is a hallmark of malignancy. Cyclin-CDK (cyclin-dependent kinase) complexes regulate this progression through the cell cycle. miRNA dysregulation promotes cell cycle progression by upregulating cyclin expression or downregulating expression of CDK inhibitors (p57, p21, *etc.*) in numerous malignancies (Figure 1). *miR-449* is downregulated in *H. pylori*-infected gastric mucosa and in gastric cancer and targets cyclin E2 and geminin. Both cyclin E2 and geminin are overexpressed in various malignancies and promote G1/S and M/G1 cell cycle progression [6]. Consequently, downregulation of *miR-449*, as occurs following *H. pylori* infection, promotes cell cycle progression and proliferation through the upregulation of cyclin E2 and geminin.

G2/M cell cycle progression and proliferation in gastric cancer cells are regulated by p42.3 [12]; *miR-29a* is significantly downregulated in gastric cancer and targets p42.3 [13] (Figure 1). Thus, the downregulation of *miR-29a* results in a reciprocal increase in p42.3 expression, promoting increased cell cycle progression and proliferation.

Both *miR-93* and *miR-106b* directly target p21, resulting in its transcriptional silencing and impairment of its tumor-suppressing activity [14]. In addition, *miR-25* targets p57, while *miR-221* and *miR-222* target p27 and p57 [15] (Figure 1). These oncogenic miRNA clusters are also significantly upregulated in gastric cancer [16]. Overexpression of most of these miRNAs results in activation of CDK2, thereby promoting G1/S phase progression.

## 4. Inhibition of Apoptosis and miRNA

**Figure 2:**
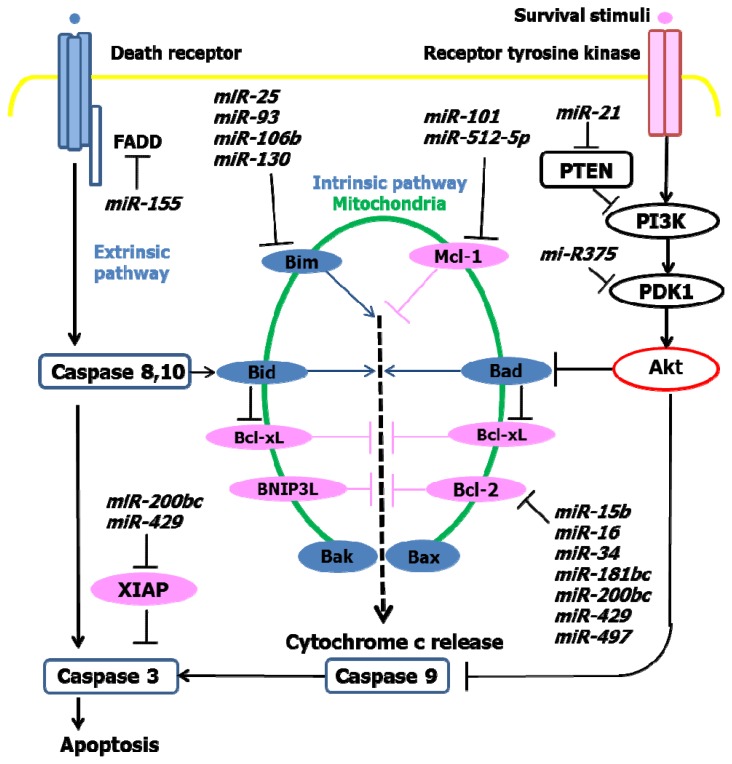
Signaling cascades that regulate the intrinsic and extrinsic pathways of apoptosis. Receptor tyrosine kinase detects survival stimuli such as growth factors and induces phosphoinositide 3-kinase (PI3K)/Akt signaling cascades that ultimately result in the inhibition of the pro-apoptotic protein, Bad. Pro-apoptotic and anti-apoptotic proteins govern the intrinsic cell death pathway, which results in the release of cytochrome *c* from the mitochondria and induction of the caspase cascade. Signaling through death receptors initiates the extrinsic pathway of apoptosis.

Evasion of apoptosis is a common feature of malignancy. Apoptosis is classifiable as either intrinsic or extrinsic pathway-dependent. The extrinsic apoptosis pathway is initiated on the cell surface through the activation of specific pro-apoptotic death receptors. Tumor necrosis factors are cytokines produced mainly by activated macrophages that bind the death receptors as their ligands. Ligand binding induces receptor clustering and the recruitment of the adaptor protein Fas-associated death domain (FADD), leading to induction of caspases and ultimately cell death. *miR-155* targets *FADD*, leading to decreased expression of this key adaptor molecule (Figure 2) [17]. Therefore, the upregulation of *miR-155* by *H. pylori* and during carcinogenesis results in the downregulation of FADD and the inhibition of apoptosis.

The intrinsic pathway is initiated within cells and hinges on the balance of activity between pro-apoptotic (e.g., Bax, Bak, Bim, BNIP3L, and Bid) and anti-apoptotic (e.g., Bcl-2, Bcl-xL, and Mcl-1) proteins from the Bcl-2 (B cell lymphoma 2) superfamily. Some miRNAs overexpressed in gastric cancer function as oncogenic miRNAs by targeting members of the pro-apoptotic proteins. *miR-25*, *miR-93*, *miR-106b*, and *miR-130* inhibit apoptosis by preventing the expression of the pro-apoptotic protein, Bim (Figure 2) [14].

Tumor suppressor miRNAs (*miR-15b*, *miR-16*, *miR-34*, *miR-181b*, *miR-181c,* and *miR-497*) target anti-apoptotic Bcl-2. These miRNA clusters are downregulated in gastric cancer cells, leading to increased expression of Bcl-2 and inhibition of apoptosis. The *miR-200bc/429* cluster is downregulated in *H. pylori*-infected gastric mucosa, and these miRNAs directly target Bcl-2 and XIAP (x-linked inhibitor of apoptosis) [18]. *miR-101* and *miR-515-5p* target Mcl-1, which are downregulated in gastric cancer, lead to increased levels of Mcl-1 and an anti-apoptotic phenotype (Figure 2).

In addition to targeting proteins directly involved in the intrinsic and extrinsic cell death pathways, miRNAs target other factors that ultimately lead to apoptosis inhibition and increased proliferation. *miR-21* targets PTEN (phosphatase and tensin homolog), a tumor suppressor and negative regulator of the PI3K/Akt signaling pathway. *miR-21* is upregulated in gastric cancer, and its overexpression shifts the balance between proliferation and apoptosis, by increasing cellular proliferation and inhibiting apoptosis.

*miR-375* targets 3-phophoinositide dependent protein kinase (PDK1), a kinase that directly phosphorylates Akt, thereby regulating the PI3K/Akt signaling pathway. Overexpression of *miR-375* reduces cell viability and *miR-375* is downregulated in gastric cancer (Figure 2) [19].

## 5. Metastasis and miRNA

Some miRNAs that are known to regulate cell cycle progression and apoptosis pathways are also involved in invasion and metastasis. *miR-181b* is aberrantly overexpressed in *H. pylori* infection and gastric cancer tissues [20]. Cell proliferation, migration, and invasion in the gastric cancer cells were significantly increased after *miR-181b* transfection, and the number of apoptotic cells was also increased. Furthermore, overexpression of *miR-181b* downregulated the protein levels of tissue inhibitor of metalloproteinase 3 (TIMP). The upregulation of *miR-181b* may play an important role in the progression of gastric cancer and *miR-181b* may be a potential molecular target for gastric cancer therapies.

*miR-218* is reduced significantly in gastric cancer tissues, *H. pylori*-infected gastric mucosa, and *H. pylori*-infected AGS cells [21]. *miR-218*, a tumor suppressor miRNA, is downregulated in gastric cancer, which correlates with increased metastasis and cancer invasion [22]. This downregulation is thought to occur through the direct targeting of roundabout homolog (ROBO1), which leads to enhanced signaling through the ROBO1 receptor. The SLIT/ROBO signaling pathway is implicated in many biological responses through regulating cell migration. Thus, disruption of this signaling cascade can result in increased invasion and metastasis. *miR-21* also targets RECK (reversion-inducing-cysteine-rich protein with kazal motifs), a tumor and metastasis suppressor that inhibits tumor metastasis and angiogenesis through modulation of matrix metalloproteinases. Recently, Li *et al.* indicated that *miR-21*, *miR-218*, and *miR-223* may be potential biomarkers for gastric cancer detection [23].

*miR-148a* is downregulated in gastric cancer. The protein interaction network regulated by *miR-148a* is associated with metastasis-related function, such as integrin-mediated signaling, cell-matrix adhesion, and blood coagulation [24]. A single miRNA can provoke a chain reaction and further affect the protein interaction network. This interactive network-based approach could provide insight into carcinogenesis.

## 6. miRNA and Anticancer Therapy

miRNAs are promising molecular targets for anticancer therapeutics in gastric cancer. Kim *et al.* reported that *miR-10b* was silenced in gastric cancer cells by promoter methylation. *miR-10b* targets the oncogene that encodes microtubule-associated protein, RP/EB family member 1. After 5-aza-2′-deoxycytidine treatment of gastric cancer cells, *miR-10b* methylation is significantly decreased, and the expression of *miR-10b* is restored. The modulation of *miR-10b* may represent a therapeutic approach for treating gastric cancer [25].

Runx3 is an important tumor suppressor that is inactivated in gastric cancer, and promoter hypermethylation of Runx3 is frequent [26]. 5-aza-2′-deoxycytidine treatment reactivates the expression of Runx3. Lai *et al.* reported that *miR-130b* expression is upregulated in gastric cancer, and this is inversely associated with Runx3 hypermethylation. *miR-130b* overexpression increases cell viability, reduces cell death, and decreases the expression of Bim in TGF-beta mediated apoptosis, subsequent to the downregulation of Runx3 protein expression. The attenuation of Runx3 protein levels by miRNA may reduce the growth suppressive potential of Runx3 and contribute to tumorigenesis [27]. Wang *et al.* reported that *miR-301a* is upregulated in gastric cancer, and directly downregulates Runx3 expression [28].

The selective cyclooxygenase-2 (COX-2) inhibitor celecoxib is a potential drug for the treatment of gastrointestinal tumors. We investigated the role of miRNAs in gastric carcinogenesis and the feasibility of a new therapeutic approach for gastric cancer [29]. miRNA microarray analysis revealed that *miR-29c* is significantly downregulated in gastric cancer tissues relative to non-tumor gastric mucosa [29]. *miR-29c* is significantly activated by celecoxib in gastric cancer cells (AGS) [29]. Celecoxib activation of *miR-29c* induces suppression of the oncogene *Mcl-1*, a target of *miR-29c* and apoptosis in gastric cancer cells. These results suggest that the downregulation of the *miR-29c* tumor suppressor plays a critical role in the progression of gastric cancer. As such, selective COX-2 inhibitors may be a clinical option for the treatment of gastric cancer via restoration of *miR-29c*.

## 7. MALT Lymphoma and miRNA

Gastric B-cell lymphoma of the mucosa-associated lymphoid tissue (MALT lymphoma) develops in the chronically inflamed mucosa of patients infected with the bacterial pathogen. In 60%–80% of these cases, the *H. pylori*-positive gastric MALT lymphoma regresses after *H. pylori* eradication. The t(11;18) (q21;q21) translocation is associated with *API2-MALT1* fusion, and this translocation responds only rarely or not at all to *H. pylori* eradication.

We previously reported that a hematopoietic-specific miRNA, *miR-142*, and an oncogenic miRNA, *miR-155*, are overexpressed in MALT lymphoma lesions [30]. *miR-142-5p* and *miR-155* suppress the proapoptotic gene *TP53INP1* as their target [30]. The expression levels of *miR-142-5p* and *miR-155* are significantly increased in MALT lymphomas that do not respond to *H. pylori* eradication [30]. The expression levels of *miR-142-5p* and *miR-155* are associated with the clinical courses of gastric MALT lymphoma cases and these miRNAs may have a potential application as novel biomarkers for gastric MALT lymphoma.

Craig *et al.* reported the strong downregulation of the putative tumor suppressor miRNA, *miR-203*, in human MALT lymphoma samples, which results from extensive promoter hypermethylation of the *miR-203* locus and coincides with the deregulation of its target, *ABL1*. Treatment of lymphoma B cells with demethylating agents leads to increased *miR-203* expression and concomitant downregulation of ABL1, confirming the effectiveness of epigenetic regulation of this miRNA. These results show that the transformation from gastritis to MALT lymphoma is epigenetically regulated by *miR-203* promoter methylation and identifies ABL1 as a novel target for treatment [31].

Although generally considered an indolent disease, MALT lymphoma may have the ability to transform into gastric diffuse large B-cell lymphoma (gDLBCL). Craig *et al.* reported that Myc overexpression is detected in 80% of gDLBCLs, but only 20% of MALT lymphomas are spotted on a tissue microarray. FoxP1 overexpression is detectible in gDLBCL, but not in gastric MALT lymphoma. *miR-34a* is downregulated in malignant lymphoma, as are the targets of *miR-34a*, Myc and FoxP1 and *miR-34a* shows strong antiproliferative properties when overexpressed in DLBCL cells. *miR-34a* replacement therapy is therefore a promising strategy in lymphoma treatment [32].

## 8. Conclusion

As we are just beginning to understand the relationship between miRNAs and gastric malignancies and the number of identified miRNA genes is increasing, there is a potential for a large number of therapeutic targets and biomarkers in this area. Further studies are necessary to investigate whether miRNA-oriented therapy is an effective strategy for the chemoprevention of gastric malignancies.

## Figures and Tables

**Table 1:** MicroRNAs (miRNAs) change in response to *Helicobacter pylori.*

miRNAs	Change	Target mRNAs	Biological process targeted
***let-7a***	**↓**	**RAB40C**	**Cell cycle progression**
		**HMGA2**	**Invasion**

*let-7b/d/e/f*	↓	HMGA2	Invasion
***miR101***	**↓**	**MCL1**	**Apoptosis**

*miR-106b*	↓	p21	Cell cycle progression
		BIM	Apoptosis

*miR-125a*	↓	ERBB2	Proliferation
***miR-141***	**↓**	**FGFR2**	**Proliferation**
*miR-200a*	↓	ZEB1, ZEB2	Epithelial to mesenchymal transition
*miR-200b/c*	↓	BCL2, XIAP	Apoptosis
***miR-203***	**↓**	**ABL1**	**Proliferation, Invasion**
*miR-204*	↓	EZR	Proliferation
***miR-218***	**↓**	**ROBO1**	**Invasion, Metastasis**

***miR-375***	**↓**	**PDK1, 12-3-3**	**Apoptosis**
		**JAK2**	**Proliferation**

*miR-429*	↓	BCL2, XIAP	Apoptosis
		MYC	Proliferation

***miR-17***	**↑**	**p21**	**Cell cycle progression**
***miR-20a***	**↑**	**p21**	**Cell cycle progression**

***miR-21***	**↑**	**PTEN**	**Proliferation**
		**RECK**	**Metastasis**

***miR-146a***	**↑**	**IRAK1, TRAF6**	**Proliferation, Immune response**
		**SMAD4**	**Apoptosis**

***miR-155***	**↑**	**IKK-ɛ, SMAD4**	**Immune response**
		**FADD, PLIα**	**Apoptosis**

***miR-223***	**↑**	**EPB41L3**	**Invasion, Metastasis**

↓: miRNA is downregulated in response to *H. pylori*; ↑: miRNA is upregulated in response to *H. pylori*; Bold indicates miRNA changes in the same way in gastric cancer.

## References

[1] Suzuki H., Iwasaki E., Hibi T. (2009). *Helicobacter pylori* and gastric cancer. Gastric Cancer.

[2] Suzuki H., Nishizawa T., Hibi T. (2010). *Helicobacter pylori* eradication therapy. Future Microbiol.

[3] Nishizawa T., Suzuki H., Nakagawa I., Minegishi Y., Masaoka T., Iwasaki E., Hibi T. (2009). Early *Helicobacter pylori* eradication restores sonic hedgehog expression in the gastric mucosa of Mongolian gerbils. Digestion.

[4] Fukase K., Kato M., Kikuchi S., Inoue K., Uemura N., Okamoto S., Terao S., Amagai K., Hayashi S., Asaka M. (2008). Effect of eradication of *Helicobacter pylori* on incidence of metachronous gastric carcinoma after endoscopic resection of early gastric cancer: An open-label, randomised controlled trial. Lancet.

[5] Saito Y., Suzuki H., Hibi T. (2009). The role of microRNAs in gastrointestinal cancers. J. Gastroenterol.

[6] Noto J.M., Peek R.M. (2011). The role of microRNAs in *Helicobacter pylori* pathogenesis and gastric carcinogenesis. Front Cell Infect Microbiol.

[7] Saito Y., Suzuki H., Tsugawa H., Suzuki S., Matsuzaki J., Hirata K., Hibi T. (2011). Dysfunctional gastric emptying with down-regulation of muscle-specific microRNAs in *Helicobacter pylori*-infected mice. Gastroenterology.

[8] Suzuki H., Nishizawa T., Tsugawa H., Mogami S., Hibi T. (2012). Roles of oxidative stress in stomach disorders. J. Clin. Biochem. Nutr.

[9] Tsugawa H., Suzuki H., Saya H., Hatakeyama M., Hirayama T., Hirata K., Nagano O., Matsuzaki J., Hibi T. (2012). Reactive oxygen species-induced autophagic degradation of *Helicobacter pylori* caga is specifically suppressed in cancer stem-like cells. Cell Host Microbe.

[10] Zhu Y., Jiang Q., Lou X., Ji X., Wen Z., Wu J., Tao H., Jiang T., He W., Wang C. (2012). MicroRNAs up-regulated by CagA of *Helicobacter pylori* induce intestinal metaplasia of gastric epithelial cells. PLoS One.

[11] Shiotani A., Uedo N., Iishi H., Murao T., Kanzaki T., Kimura Y., Kamada T., Kusunoki H., Inoue K., Haruma K. (2012). *H. pylori* eradication did not improve dysregulation of specific oncogenic miRNAs in intestinal metaplastic glands. J. Gastroenterol.

[12] Xu X., Li W., Fan X., Liang Y., Zhao M., Zhang J., Tong W., Wang J., Yang W., Lu Y. (2007). Identification and characterization of a novel p42.3 gene as tumor-specific and mitosis phase-dependent expression in gastric cancer. Oncogene.

[13] Cui Y., Su W.Y., Xing J., Wang Y.C., Wang P., Chen X.Y., Shen Z.Y., Cao H., Lu Y.Y., Fang J.Y. (2011). MiR-29a inhibits cell proliferation and induces cell cycle arrest through the downregulation of p42.3 in human gastric cancer. PLoS One.

[14] Kan T., Sato F., Ito T., Matsumura N., David S., Cheng Y., Agarwal R., Paun B.C., Jin Z., Olaru A.V. (2009). The miR-106b-25 polycistron, activated by genomic amplification, functions as an oncogene by suppressing p21 and Bim. Gastroenterology.

[15] Kim Y.K., Yu J., Han T.S., Park S.Y., Namkoong B., Kim D.H., Hur K., Yoo M.W., Lee H.J., Yang H.K. (2009). Functional links between clustered microRNAs: Suppression of cell-cycle inhibitors by microRNA clusters in gastric cancer. Nucleic Acids Res.

[16] Volinia S., Calin G.A., Liu C.G., Ambs S., Cimmino A., Petrocca F., Visone R., Iorio M., Roldo C., Ferracin M. (2006). A microRNA expression signature of human solid tumors defines cancer gene targets. Proc. Natl. Acad. Sci. USA.

[17] Xiao B., Liu Z., Li B.S., Tang B., Li W., Guo G., Shi Y., Wang F., Wu Y., Tong W.D. (2009). Induction of microRNA-155 during *Helicobacter pylori* infection and its negative regulatory role in the inflammatory response. J. Infect Dis.

[18] Zhu W., Xu H., Zhu D., Zhi H., Wang T., Wang J., Jiang B., Shu Y., Liu P. (2012). miR-200bc/429 cluster modulates multidrug resistance of human cancer cell lines by targeting BCL2 and XIAP. Cancer Chemother. Pharmacol.

[19] Tsukamoto Y., Nakada C., Noguchi T., Tanigawa M., Nguyen L.T., Uchida T., Hijiya N., Matsuura K., Fujioka T., Seto M. (2010). MicroRNA-375 is downregulated in gastric carcinomas and regulates cell survival by targeting PDK1 and 14-3-3zeta. Cancer Res.

[20] Guo J.X., Tao Q.S., Lou P.R., Chen X.C., Chen J., Yuan G.B. (2012). miR-181b as a potential molecular target for anticancer therapy of gastric neoplasms. Asian Pac. J. Cancer Prev.

[21] Gao C., Zhang Z., Liu W., Xiao S., Gu W., Lu H. (2010). Reduced microRNA-218 expression is associated with high nuclear factor kappa B activation in gastric cancer. Cancer.

[22] Tie J., Pan Y., Zhao L., Wu K., Liu J., Sun S., Guo X., Wang B., Gang Y., Zhang Y. (2010). MiR-218 inhibits invasion and metastasis of gastric cancer by targeting the Robo1 receptor. PLoS Genet.

[23] Li B.S., Zhao Y.L., Guo G., Zhu E.D., Luo X., Mao X.H., Zou Q.M., Yu P.W., Zuo Q.F., Li N. (2012). Plasma microRNAs, miR-223, miR-21 and miR-218, as novel potential biomarkers for gastric cancer detection. PLoS One.

[24] Tseng C., Lin C.C., Chen C.N., Huang H.C., Juan H.F. (2011). Integrative network analysis reveals active microRNA and their functions in gastric cancer. BioMed. Central Syst. Biol.

[25] Kim K., Lee H.C., Park J.L., Kim M., Kim S.Y., Noh S.M., Song K.S., Kim J.C., Kim Y.S. (2011). Epigenetic regulation of microRNA-10b and targeting of oncogenic MAPRE1 in gastric cancer. Epigenetics.

[26] Suzuki M., Suzuki H., Minegishi Y., Ito K., Nishizawa T., Hibi T. (2010). *H. pylori*-eradication therapy increases runx3 expression in the glandular epithelial cells in enlarged-fold gastritis. J. Clin. Biochem. Nutr.

[27] Lai K.W., Koh K.X., Loh M., Tada K., Subramaniam M.M., Lim X.Y., Vaithilingam A., Salto-Tellez M., Iacopetta B., Ito Y. (2010). MicroRNA-130b regulates the tumour suppressor RUNX3 in gastric cancer. Eur. J. Cancer.

[28] Wang M., Li C., Yu B., Su L., Li J., Ju J., Yu Y., Gu Q., Zhu Z., Liu B. (2013). Overexpressed miR-301a promotes cell proliferation and invasion by targeting RUNX3 in gastric cancer. J. Gastroenterol..

[29] Saito Y., Suzuki H., Imaeda H., Matsuzaki J., Hirata K., Tsugawa H., Hibino S., Kanai Y., Saito H., Hibi T. (2013). The tumor suppressor microRNA-29c is downregulated and restored by celecoxib in human gastric cancer cells. Int. J. Cancer.

[30] Saito Y., Suzuki H., Tsugawa H., Imaeda H., Matsuzaki J., Hirata K., Hosoe N., Nakamura M., Mukai M., Saito H. (2012). Overexpression of miR-142-5p and miR-155 in Gastric Mucosa-Associated Lymphoid Tissue (MALT) lymphoma resistant to *Helicobacter pylori* eradication. PLoS One.

[31] Craig V.J., Cogliatti S.B., Rehrauer H., Wundisch T., Muller A. (2011). Epigenetic silencing of microRNA-203 dysregulates ABL1 expression and drives *Helicobacter*-associated gastric lymphomagenesis. Cancer Res.

[32] Craig V.J., Cogliatti S.B., Imig J., Renner C., Neuenschwander S., Rehrauer H., Schlapbach R., Dirnhofer S., Tzankov A., Muller A. (2011). Myc-mediated repression of microRNA-34a promotes high-grade transformation of B-cell lymphoma by dysregulation of FoxP1. Blood.

